# Sulfasalazine‐Induced Urinary Normetanephrine Elevation Mimicking Recurrent Phaeochromocytoma—A Case Report

**DOI:** 10.1155/crie/6661577

**Published:** 2026-02-16

**Authors:** Maria Hadjicosti, Anastasia Papapostolou, Michail Papoulas, Evdoxia Poulianiti, Nikoleta Christodoulidou, Michalis K. Picolos

**Affiliations:** ^1^ Department of Radiology, Nicosia General Hospital, Nicosia, Cyprus, moh.gov.cy; ^2^ Alithias Endocrinology Center, Nicosia, Cyprus; ^3^ Department of Medicine and Dentistry European University, Nicosia, Cyprus; ^4^ Department of General Surgery, El Greco Medical Centre, Nicosia, Cyprus; ^5^ Department of Medicine and Dentistry Queen Mary University of London, London, UK

**Keywords:** case report, liquid chromatography tandem mass spectrometry, normetanephrine, phaeochromocytoma, sulfasalazine

## Abstract

Phaeochromocytomas and paragangliomas (PPGLs) are catecholamine‐secreting neuroendocrine tumours (NETs) of the adrenal medulla and autonomic nervous system. Early recognition and management is critical given their potential morbidity and mortality. For this reason, stand‐alone screening investigations rely on a low diagnostic threshold, achieving high sensitivity at the relative cost of specificity. Following diagnosis, the only curative option is surgical removal of the tumour. Similar investigations are employed for postoperative surveillance. Persistent urinary normetanephrine elevation after curative phaeochromocytoma resection is rare and may lead to unnecessary investigations and anxiety. Our case highlights a previously underreported cause—analytical interference from sulfasalazine—and underscores the importance of considering medication effects in postoperative biochemical surveillance. We hereby present a 73‐year‐old Caucasian woman with a history of rheumatoid arthritis treated with sulfasalazine, hypertension, type 2 diabetes and hypothyroidism who exhibited persistently and significantly elevated urine normetanephrine, up to 15 months following successful surgical resection of a phaeochromocytoma. This was secondary to long‐term sulfasalazine use, causing gross interference with laboratory urine normetanephrine analysis without interfering with the serum normetanephrine value. Discontinuation of sulfasalazine normalised the urine normetanephrine results. This case identifies sulfasalazine as a clinically relevant source of urine normetanephrine assay interference, reinforcing the need for careful interpretation of biochemical diagnostic or surveillance results.

## 1. Background

Phaeochromocytomas are neuroendocrine tumours (NETs) originating from chromaffin cells within the adrenal medulla. By virtue of their functional tumour nature, they secrete catecholamines, acting as neurotransmitters and hormones of the sympathetic nervous system.

Phaeochromocytomas escape the natural negative feedback control, causing unregulated catecholamine secretion. This commonly manifests with a pathognomonic triad of episodic palpitations, headaches and sweating, among other symptoms [1].

Diagnosis relies on biochemical evidence of catecholamine excess with superimposed radiological and histological (post‐resection) evidence localising the lesion to the adrenal medulla and chromaffin cells. Genetic testing is also vital due to the high prevalence of germline mutations in susceptibility genes [2].

Due to the potentially fatal side effects of a perpetuated sympathetic response, adequate management is pertinent. Surgical resection is the only curative option, and post‐surgical follow‐up is advised to ensure lack of residual/recurrent pathology [3].

Biochemical investigations exploit a spiral approach with initial screening investigations involving the measurement of plasma‐free metanephrine and normetanephrine as well as urinary metanephrine and normetanephrine [4]. Numerous factors influence the reliability of such tests due to the impact on specificity, sensitivity and predictive values [5].

Given the gravity of a phaeochromocytoma and paraganglioma (PPGL) diagnosis, there is emphasis towards a low diagnostic threshold for the initial screening investigations, to maximise sensitivity and minimise the combined risk of Type II errors (false negatives) [5].

Our case highlights persistent elevation of urinary normetanephrines for 15 months following curative resection of a phaeochromocytoma, caused not by recurrent disease but by analytical interference from sulfasalazine. While elevated urinary normetanephrine is commonly used as a sensitive marker for catecholamine‐secreting tumours, significant medication‐induced false positives are rare and underreported. Recognition of such interference is essential to avoid unnecessary investigations, patient anxiety and misinterpretation of postoperative surveillance results. Previous literature has described drug‐related interference in initial diagnosis, but none, to our knowledge, has addressed this phenomenon in post‐resection follow‐up [6–8]. This case emphasises the importance of integrating biochemical results, imaging and thorough medication review into the postoperative management of phaeochromocytoma.

## 2. Case Presentation

A 73‐year‐old Caucasian female presented to our outpatient clinic for re‐evaluation due to persistently elevated 24 h urine normetanephrines, 15 months after successful resection of a phaeochromocytoma.

Past medical history included Hashimoto thyroiditis and hypothyroidism, type 2 diabetes mellitus, hypertension, carotid artery stenosis, hyperlipidaemia, atrial fibrillation, rheumatoid arthritis, gastritis and vitamin D deficiency. She had no significant family history, and her medications were: levothyroxine, metformin, sitagliptin, olmesartan, hydrochlorothiazide, amlodipine, bisoprolol, atorvastatin, flecainide, rivaroxaban, sulfasalazine, pantoprazole and vitamin D3.

Prior to her initial phaeochromocytoma diagnosis, her symptomatology involved an episodic and recurrent history of raised blood pressure despite a maximal dose of three antihypertensive medications, as well as episodic palpitations. Postoperatively, her symptoms had completely resolved despite persistent biochemical derangement.

An initial diagnostic work‐up prior to her surgery was suggestive of PPGL based on the biochemical evidence of significantly raised 24 h urine normetanephrine (1766.0 and 2210.0 μg/24 h [μg/24 h]; 9.6 and 12.1 micromol/24 h [μmol/24 h]; reference range [RR]: 52–341 μg/24 h; 0.28–1.85 μmol/24 h) on repeat testing using liquid chromatography tandem mass spectrometry (LC‐MS/MS) (Table 1). This was supported by a compliant history of recurrent episodes of palpitations and hypertension. All other endocrine investigations were in range, but the lack of concomitant urine metanephrine elevation was puzzling (Table 1).

**Table 1:** Preoperative and postoperative investigations of catecholaminergic metabolites.

	3 months pre‐surgery	2 months pre‐surgery	2 months after surgery	3 months after surgery	8 months after surgery	15 months after surgery	Off sulfasalazine–16 months after surgery	On sulfasalazine–25 months after surgery	Measuring units	Reference range
24 h urine metanephrine	39.1	98.0	30.0	155.0	170.0	36.4	55.0	—	μg/24 h	52–341
24 h urine normetanephrine	1766.0	2210.0	768.0	2980.0	1970.0	8000.0	243.0	—	μg/24 h	148–560
24 h urine creatinine	—	—	1147.0	2179.0	1371.0	1250.0	1201.0	—	mg/24 h	800–1800
24 h urine volume	2200	1900	3100	2400	2050	2450	1850	—	mL	—
Plasma‐free metanephrine	—	—	—	—	—	15.50	<10.00	<10.00	ng/L	<88.00
Plasma‐free normetanephrine	—	—	—	—	—	178.00	141.00	27.20	ng/L	<200
Serum chromogranin A	—	—	—	487.00	518.00	—	—	—	ng/mL	<98.00

Following the above results, a CT scan of the abdomen was performed revealing an 11 × 10 × 10 millimetre lesion in the left adrenal gland of 33 HU on the pre‐contrast images with mild enhancement in both arterial and venous post‐contrast imaging (Figure 1A, B). No lymphadenopathy was evident.

Figure 1(A) Preoperative CT imaging showing the left adrenal lesion in a cross‐sectional snapshot of the arterial phase of contrast enhancement. (B) Preoperative CT imaging showing the left adrenal lesion in a coronal snapshot of the venous phase of contrast enhancement.

**Figure:**
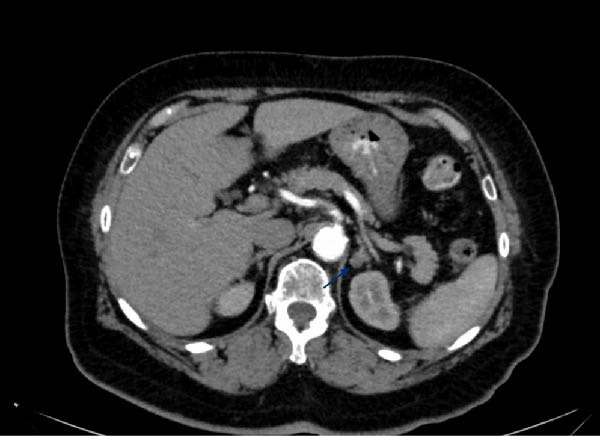
(A)

**Figure:**
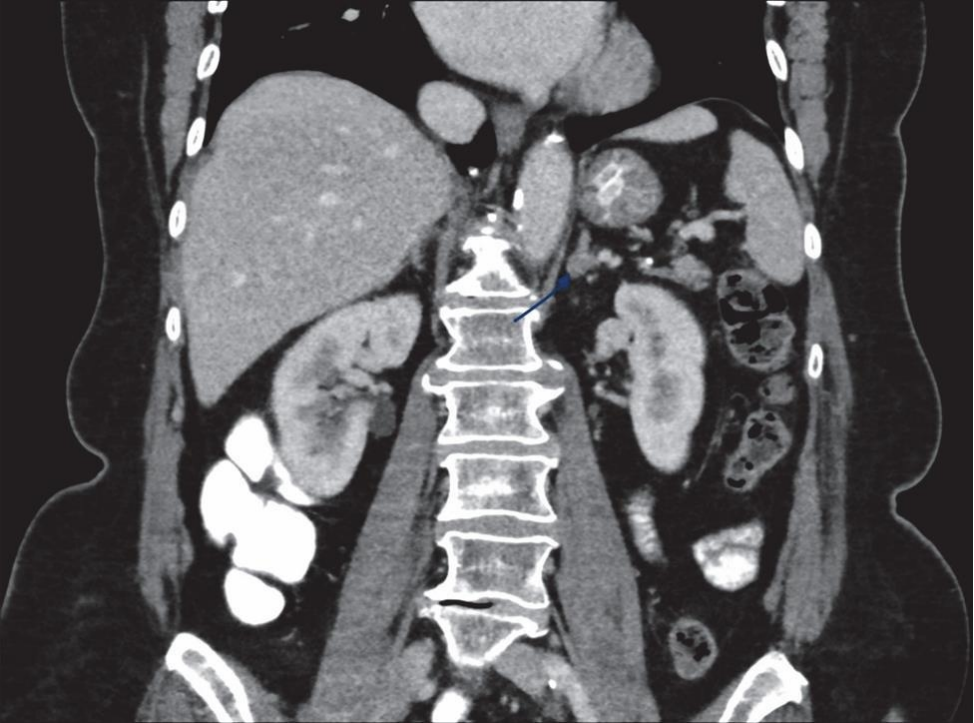
(B)

Additionally, genetic testing for the phaeochromocytoma gene panel was undertaken using genomic DNA from blood samples, with no clinically significant sequence variants detected.

The patient had a complete surgical resection of the lesion by means of laparoscopic left adrenalectomy. Histopathological analysis showed a circumscribed tumour with clear margins arising from the medulla, consisting of large pleomorphic basophilic cells arranged in nests with perivascular growth. Tumour cells were strongly and diffusely positive for chromogranin. Mitotic figures were sparse and there were no atypical mitotic figures. The lesion was classified as a Phaeochromocytoma of the Adrenal Gland Scaled Score (PASS) of 0 with very low probability of malignancy.

Postoperatively, she was still maintained on three antihypertensive medications, with the blood pressure being well controlled and no further symptomatology.

## 3. Investigation

Follow‐up biochemistry revealed persistently raised 24 h urinary normetanephrine (up to 18‐fold) on multiple occasions over the span of 15 months (lowest level: 768 μg/24 h [3.6 μmol/24 h]; highest level: 8000 μg/24 h [37.5 μmol/24 h]), RR: 52–341 μg/24 h; 0.28–1.85 μmol/24 h, with low‐normal 24 h urinary metanephrine (Table 1). Chromogranin A was also raised (487 ng/mL, [RR: <98 ng/mL]). Repeat testing in different laboratories (using LC‐MS/MS) for urine metanephrine and normetanephrine was consistent. Table 1 summarises the relevant biochemistry.

Secondary to this, there were concerns of possible residual/recurrent tumour burden, ectopic PPGL and/or metastatic lesions. A magnetic resonance imaging (MRI) scan was normal, and subsequent radionuclide ^18^F‐SiFAlin‐TATE PET/CT scan showed no pathological somatostatin‐expressing lesions.

Given the asymptomatic clinical picture postoperatively, along with the negative radiological evidence of foci of disease and the negative nuclear medicine screening, alternative explanations were investigated. A thorough medication review identified two medications as potential culprits for analytical interference of results. Pantoprazole, a proton pump inhibitor, has been established in literature to be a cause of increased chromogranin A levels [9, 10]. Sulfasalazine, an anti‐inflammatory medication used in rheumatoid arthritis, has previously been associated with raised urinary normetanephrine [6–8] .

The hypothesis that the spurious results were secondary to medication interference was supported by normal plasma metanephrine (15.50 ng/L, [RR: <88.0]; Table 1) and normetanephrine (178.00 ng/L, [RR: <200.00]; Table 1) performed even during sulfasalazine administration despite the concomitant 24 h urine elevation. Furthermore, suspending sulfasalazine intake for 2 weeks resulted in normalisation of the 24 h urine normetanephrine (243.00 μg/24 h, [RR: 52–349]), confirming sulfasalazine interference isolated to urine normetanephrine with no effect on plasma sample concentration.

## 4. Discussion

For PPGL, the axiom is that initial investigations should harbour a high sensitivity and negative predictive value. The associated risk of higher false positive rates can be mitigated by a combination of strong pretest screening, with only patients with a high pretest probability of disease being offered further testing given the rarity of the diagnosis (prevalence <0.05% in the general population; 0.2%–0.6% in the hypertensive population) [11], considering the magnitude of deranged biochemistry and subsequent radiological and histological evidence of disease. This concept, however, relies on strict long‐term surveillance for those low‐risk individuals with regard to worrisome phenotypic changes in keeping with a phaeochromocytoma diagnosis. This approach minimises the risk of false negatives in both the high‐ and low‐risk individuals but has implications on the need for long‐term compliance with testing.

Precise characterisation of the diagnostic potential of different investigations has been historically a notorious task due to the multidimensional impact of confounding variables in the pre‐analytical, analytical and post‐analytical domains. Nonetheless, a recent large prospective study has shown superiority of plasma‐free compared to urine‐free metabolites with regard to sensitivity (96.7% vs. 89.6%, *p*  < 0.05) with similar specificities (92.8%) for the high pretest probability group. No significant differences were shown in the low pretest probability group [12]. There is, however, a paucity of robust evidence about optimal testing regimes, and future research should focus efforts on optimising testing protocols for PPGL.

Measurement of urine and plasma concentration of catecholamine metabolites forms the basis of a PPGL diagnosis. Measurement of the metabolites instead of catecholamines themselves is more reliable because these are continuously being released systemically by the tumour, resulting in a sustained pool, contrary to the episodic release of catecholamines. Limitations to interpretation still exist however, including the influence of pre‐analytical factors (medications, patient preparation, positioning and sample stability), analytical factors (assay) and post‐analytical factors (RRs and subjective result interpretation) [5].

Use of LC‐MS/MS over high‐performance liquid chromatography with electrochemical detection (HPLC‐ECD) was shown to provide superior analytical specificity and sensitivity with lower analytical interference [13].

Following convincing biochemical evidence of pathology, imaging helps localise the lesion. A CT scan of the chest, abdomen and pelvis is recommended as first line due to its high spatial resolution [14]. MRI and functional imaging are recommended on suspicion of metastasis. Genetic testing aims to identify inherited traits for genetic counselling of family members. The ultimate diagnosis comes from post‐excision biopsy and histological analysis.

Our case refers to a patient with a low pretest probability and low positive likelihood ratio of residual/recurrent tumour burden given: 1. the successful surgical removal with clear margins, 2. the lack of evidence of metastatic potential on histology, 3. the negative postoperative imaging and 4. the complete resolution of symptoms. Despite this, investigations were undertaken as part of the postoperative follow‐up protocol. On repeat testing under different laboratories, urinary normetanephrine was persistently and significantly elevated, and in combination with a raised chromogranin A, this raised concerns and led to unnecessary investigations and patient anxiety. However, on thorough medication review, sulfasalazine and pantoprazole were identified as potential interfering factors in result interpretation. It is also possible that even on first presentation, the patient had a non‐biochemically detectable phaeochromocytoma, given that urinary metanephrine (more commonly raised in phaeochromocytomas) was normal and the size of the tumour was less than 2 cm [15]. Temporary discontinuation of sulfasalazine for 2 weeks normalised the urine normetanephrine.

Sulfasalazine is a prodrug metabolised in the gut to 5‐aminosalicylic acid (mesalamine) and sulphapyridine. The byproducts of metabolism are partly excreted in the urine and can interfere with the analysis of urine samples, more so than serum samples. Analytic interference can originate from multiple sources, including the possibility of drug metabolites forming a peak on the chromatogram in close proximity to the urinary normetanephrine peak, resulting in signal misinterpretation [16, 17]. The exact identity of the interfering compound(s), however, is uncertain. This phenomenon appears to have been encountered in the past with case reports relaying similar analytical interferences in patients with adrenal lesions but to our knowledge not in cases of surveillance after a phaeochromocytoma resection [6–8].

This case report highlights that elevated urinary normetanephrine concentration, even when assessed using LC‐MS/MS, may reflect medication‐related analytical interference rather than recurrent disease in patients receiving sulfasalazine. The underlying mechanism is likely attributable to urinary excretion of sulfasalazine metabolites that produce overlapping chromatographic peaks. Careful review of the patient’s medication history, coupled with confirmatory plasma normetanephrine measurement and potentially short‐term sulfasalazine discontinuation, if possible, is essential to prevent misdiagnosis and avoid unnecessary clinical investigations.

## Author Contributions

All authors made individual contributions to authorship. Michalis K. Picolos, Maria Hadjicosti, Michail Papoulas were involved in the diagnosis and management of this patient and patient consent. Michalis K. Picolos was involved in manuscript production and submission. All six authors were involved in writing the case report. Michalis K. Picolos had full access to all of the data in this study and takes complete responsibility for the integrity of the data and the accuracy of the data analysis.

## Funding

No public or commercial funding.

## Disclosure

All authors have read and approved the final version of the manuscript.

## Consent

Signed informed consent was obtained directly from the patient.

## Conflicts of Interest

The authors declare no conflicts of interest.

## Patient Perspective

The patient consistently demonstrated confidence and trust in her medical teams. She reported, however, increased anxiety and fear during the period of undergoing repeated investigations. This was followed by relief and reassurance upon learning that her elevated urine normetanephrine results were related to medication interference rather than recurrent disease. She willingly provided informed consent and supported publication to raise awareness.

## Data Availability

The data that support the findings of this study are available from the corresponding author upon reasonable request.
